# Supplementary material for: Methylation-based signature to distinguish indolent and aggressive prostate cancer
Source: Biol Open. 2025 Dec 23;14(12):bio062281. doi: 10.1242/bio.062281 (PMC12772133; doi:10.1242/bio.062281)
Supplement: Supplementary information [file biolopen-14-062281-s1.pdf]

## Supplementary Materials and Methods

When developing the risk-score, we fit the low-risk and high-risk samples to a logistic regression model and kept the coefficient. The sum of the times result of the methylation value and corresponding coefficient on each DMR was defined as the risk-score which was listed in Eqn S2 and could be abstracted as Eqn S2.

$$\text{Risk Score} = \sum_{i=1}^{13} \text{Methy}_i \times \beta_i - 10.64774 \qquad \text{(S1)}$$

$$\begin{aligned} Risk\ Score = \ & Methy_{\text{chr1\_27179908\_27180212}} \times (-44.849149) \\ & + Methy_{\text{chr11\_23553837\_23554122}} \times (-16.874881) \\ & + Methy_{\text{chr11\_96194062\_96194260}} \times (58.541466) \\ & + Methy_{\text{chr12\_122710046\_12271028}} \times (-3.442465) \\ & + Methy_{\text{chr12\_74063892\_74064278}} \times (1.653324) \\ & + Methy_{\text{chr12\_9968250\_9968453}} \times (-44.787969) \\ & + Methy_{\text{chr18\_52123037\_52123163}} \times (27.943572) \\ & + Methy_{\text{chr2\_162888607\_162888706}} \times (-20.971386) \\ & + Methy_{\text{chr3\_183997456\_183997736}} \times (69.454352) \\ & + Methy_{\text{chr3\_95125888\_95125987}} \times (17.558902) \\ & + Methy_{\text{chr4\_142401049\_142401283}} \times (8.664591) \\ & + Methy_{\text{chr9\_101433398\_101433510}} \times (6.722558) \\ & + Methy_{\text{chr9\_104670039\_104670489}} \times (15.541526) \\ & + Methy_{\text{chr9\_1444688\_1444802}} \times (-36.581597) \qquad \text{(S2)} \\ & - 10.64774 \end{aligned}$$

Biology Open • Supplementary information

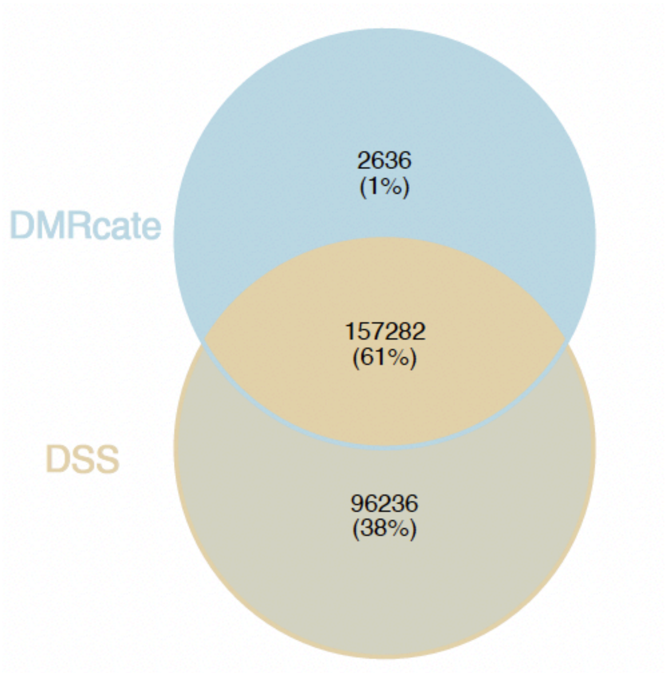


**Fig. S1.** Venn plot of DMR detection source

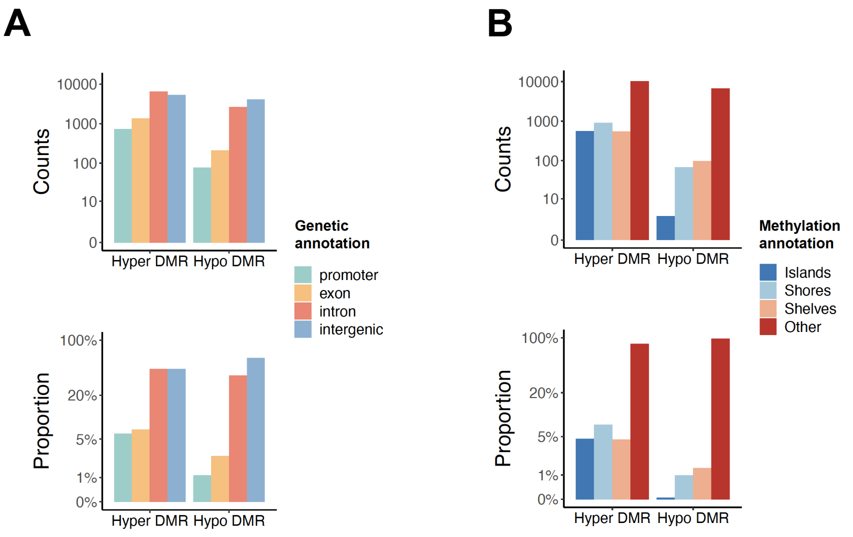


**Fig. S2.** (A) Genetic and (B) methylation location annotation of hyper and hypo DMRs.

Biology Open • Supplementary information

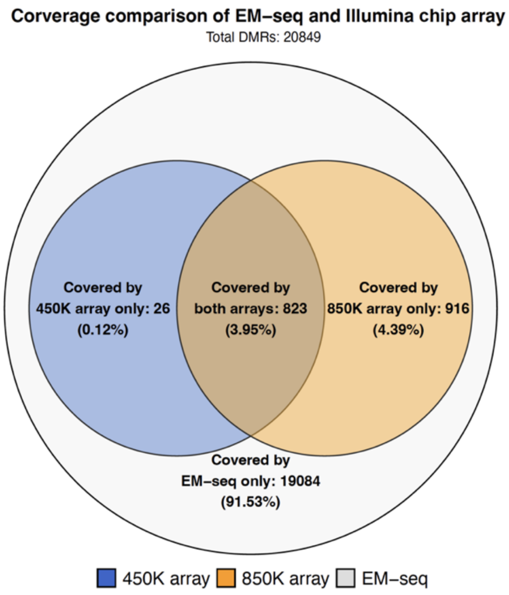


**Fig. S3.** Coverage comparison of EM−seq and Illumina chip array

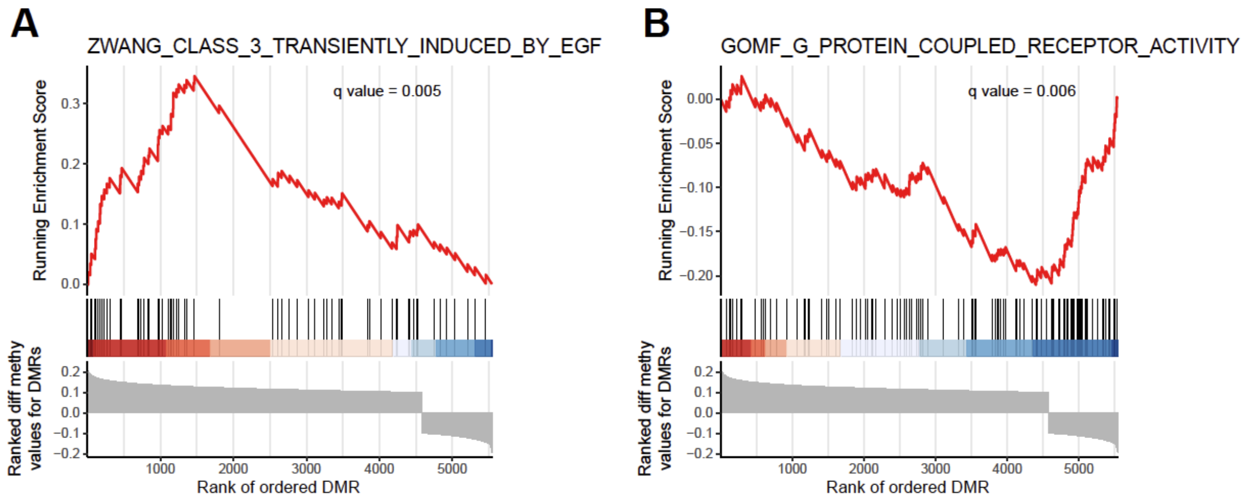


**Fig. S4.** Two significantly enriched gene sets in GSEA. (A) Class III of genes transiently induced by EGF. (B) Combining with an extracellular signal and transmitting the signal across the membrane by activating an associated G-protein; promotes the exchange of GDP for GTP on the alpha subunit of a heterotrimeric G-protein complex.

Biology Open • Supplementary information

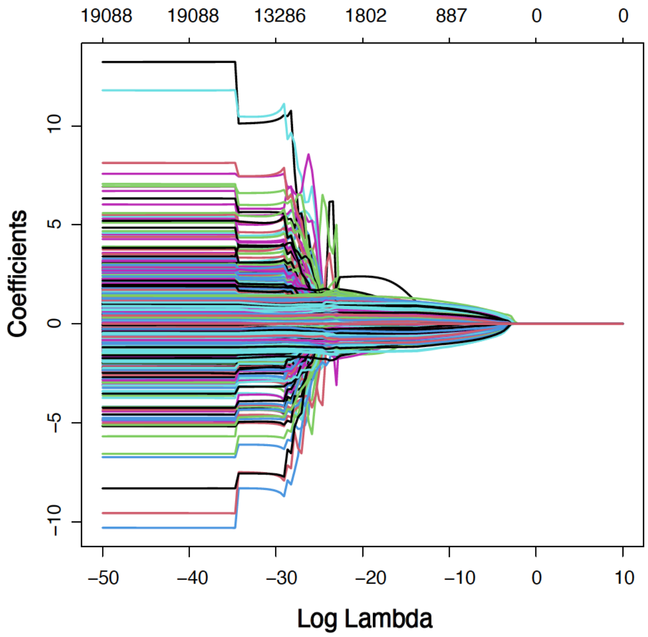


**Fig. S5.** A typical ENR process for rapid DMR screening via lambda parameter adjustment, showing the relationship of lambda , number of remaining variable, and relevant coefficients in ENR.

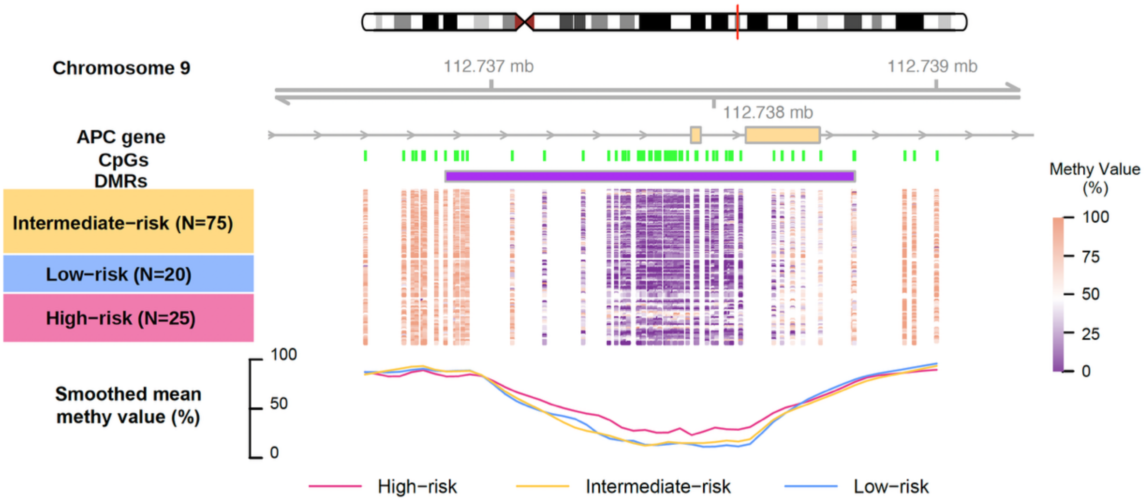


**Fig. S6.** Genome browser showing the methylation level of DMR within the range of gene APC in each sample and risk group. Smoothed mean methylation values of low-risk (light blue), intermediate-risk (orange), and high-risk (deep pink) were also showed.

Biology Open • Supplementary information

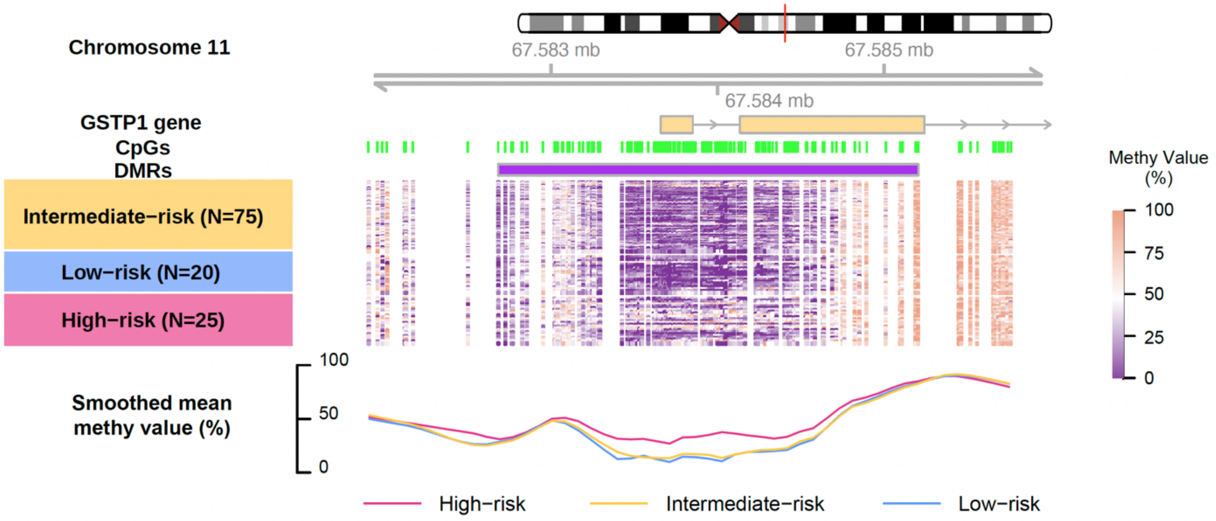


**Fig. S7.** Genome browser showing the methylation level of DMR within the range of gene GSTP1 in each sample and risk group. Smoothed mean methylation values of low-risk (light blue), intermediate-risk (orange), and high-risk (deep pink) were also showed.

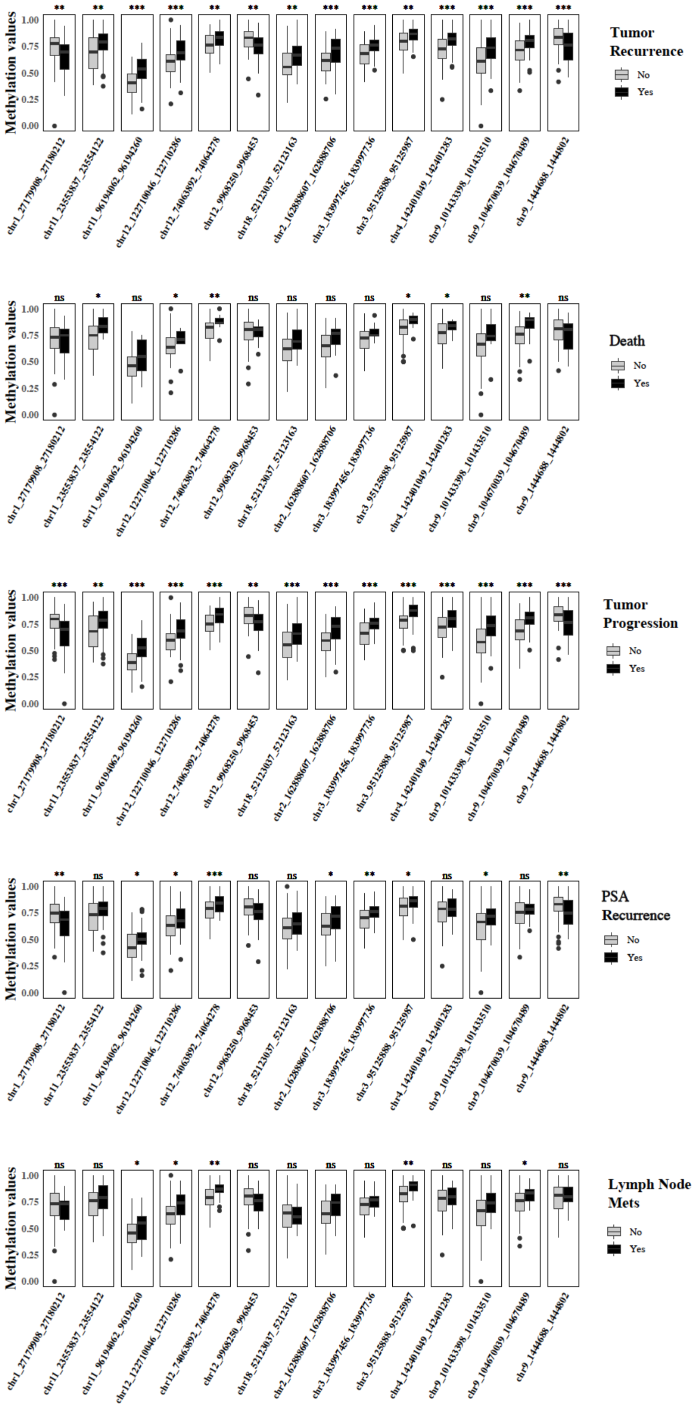


**Fig. S8.** Methylation comparison of the 14 DMRs within different clinical outcomes (Wilcoxon signed-rank test was applied to get the significance: ns: p-value > 0.05, *: 0.05 > p-value < 0.01, **: 0.01 > p-value > 0.001, ***: 0.001 > p-value).

Biology Open • Supplementary information

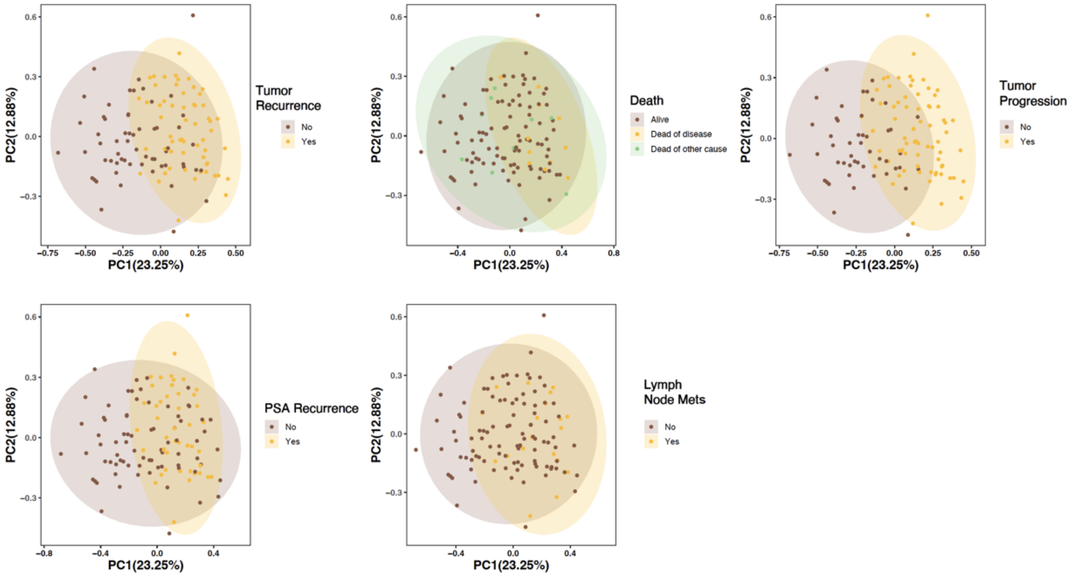


**Fig. S9.** PCA of 14-DMR signature with different clinical outcome annotation.

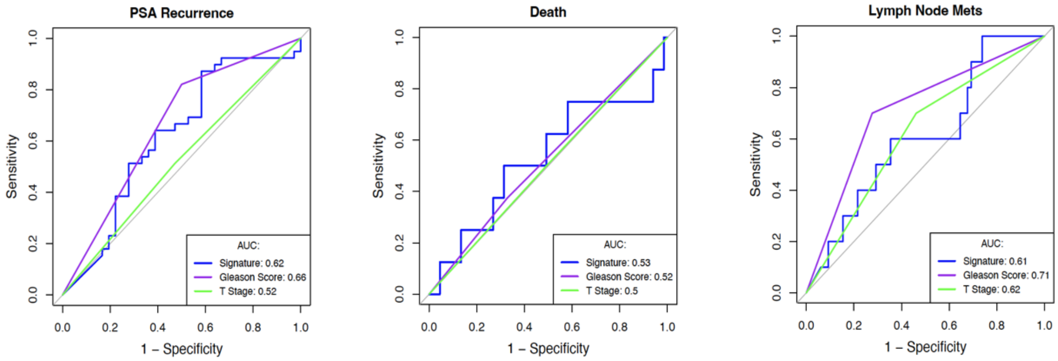


**Fig. S10 .** The results of prognostic prediction at different clinical outcomes.
